# Case report: Successful therapy with azacitidine for acute myeloid leukemia with NUP98::RARG resembling acute promyelocytic leukemia

**DOI:** 10.3389/fonc.2024.1460557

**Published:** 2024-09-04

**Authors:** Zhichen Wei, Linlin Shao, Shuqian Xu, Xiaolin Zhang, Lin Wang, Ping Qin, Qiang Song, Ming Hou, Yan Shi

**Affiliations:** ^1^ Department of Hematology, Qilu Hospital of Shandong University, Jinan, China; ^2^ Shandong Provincial Key Laboratory of Immunohematology, Qilu Hospital, Cheeloo College of Medicine, Shandong University, Jinan, China

**Keywords:** acute myeloid leukemia, acute promyelocytic leukemia, all-trans retinoic acid, nucleoporin 98-retinoic acid receptor gamma, azacitidine

## Abstract

We report a case of acute myeloid leukemia (AML) with retinoic acid receptor gamma (RARG) rearrangement, exhibiting clinical, morphological, and immunophenotypic features similar to classic acute promyelocytic leukemia (APL). RNA sequencing analysis of the patient’s bone marrow samples revealed the presence of nucleoporin 98 (NUP98)-RARG caused by translocation. AML with RARG rearrangement is insensitive to all-trans retinoic acid (ATRA) and arsenic trioxide. The patient received azacitidine therapy after failing ATRA and standard 3 + 7 therapy (idarubicin and cytarabine) and achieved complete remission. Conclusively, this acute myeloid leukemia subtype may benefit from azacitidine.

## Introduction

AML is a highly heterogeneous clonal disease of malignant hematopoietic stem cells, and APL is a distinct AML subtype. Patients with APL carrying the promyelocytic leukemia-retinoic acid receptor alpha (PML::RARA) fusion gene caused by the chromosomal translocation t(15;17)(q22;q21) are sensitive to ATRA and ATO treatments (1).

AML with nucleoporin 98- retinoic acid receptor gamma (NUP98-RARG) is identical to APL in terms of morphology, immunophenotype, and clinical manifestations but lacks the t(15;17)(q22;q21)/PML-RARA fusion (2). RARG, a member of the nuclear receptor superfamily, shares high homology with the retinoic acid receptors RARA and RARB, which are involved in retinoid signaling (3). An increasing number of patients with NUP98::RARG, PML::RARG, CPSF6::RARG, NPM1::RARG, and HNRNPc::RARG fusions are being reported. Nearly all AML cases demonstrate resistance to ATRA, and some patients show sensitivity to an AML chemotherapy regimen. Although chemotherapy may be effective as an alternative therapy in some patients, the prognosis of NUP98-RARG AML remains inferior to that of typical APL (4). This is the first case in which azacitidine therapy after the failure of idarubicin and cytarabine (IA) and ATRA regimens resulted in favorable outcomes.

## Case presentation

In May 2023, a 32-year-old man presented to a hospital in Jinan, Shandong Province, China with fever and cough. Laboratory blood tests showed hemoglobin level, 96 g/L (reference range, 130–175 g/L); platelets, 21 × 10^9^/L (reference range, 125–350 × 10^9^/L); white blood cell count, 72.06 × 10^9^/L (reference range, 3.5–9.5 × 10^9^/L); and D-dimer concentration, 76.34 mg/L (reference range, 20.00–40.00 mg/L). The bone marrow (BM) aspirate morphology showed hypercellular BM with 97% abnormal promyelocytic granulocytes. Auer bodies were observed in some cells. The reverse transcription-polymerase chain reaction (RT–PCR) and fluorescence *in situ* hybridization (FISH) failed to detect the PML::RARA transcript in the BM. The karyotype analysis revealed 46, XY, t(11;12) (p15;q14) (10)/45, idem, -Y (7)/46, idem, -Y, +?8 (3). Hence, the patient was diagnosed with AML and was administered a treatment regimen of 20 mg all-trans retinoic acid (ATRA) twice a day in combination with IA induction (idarubicin 10 mg/m^2^ on days 1–3, 100 mg/m^2^ cytarabine on days 1–7).

**Figure 1:**
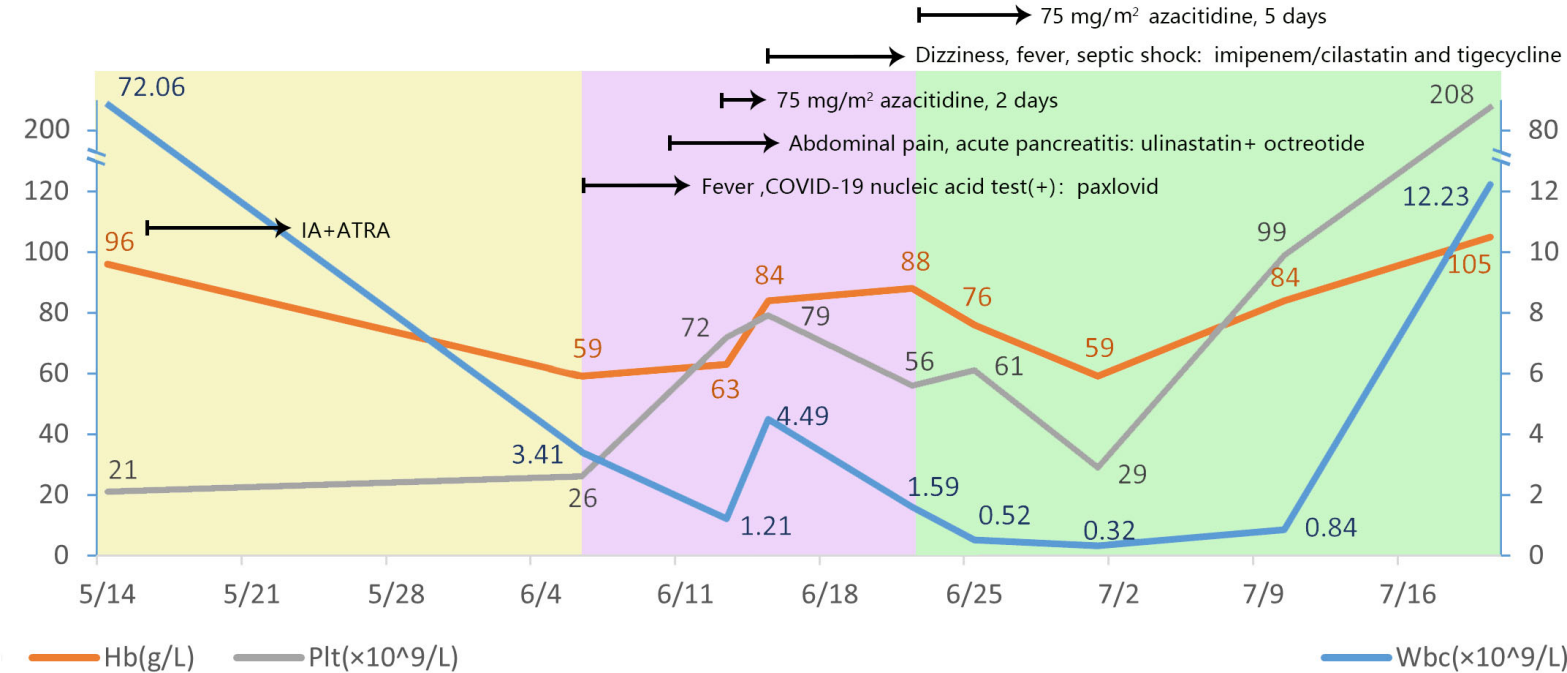
Changes in trilineage count in patients from onset to complete remission.

**Figure 2:**
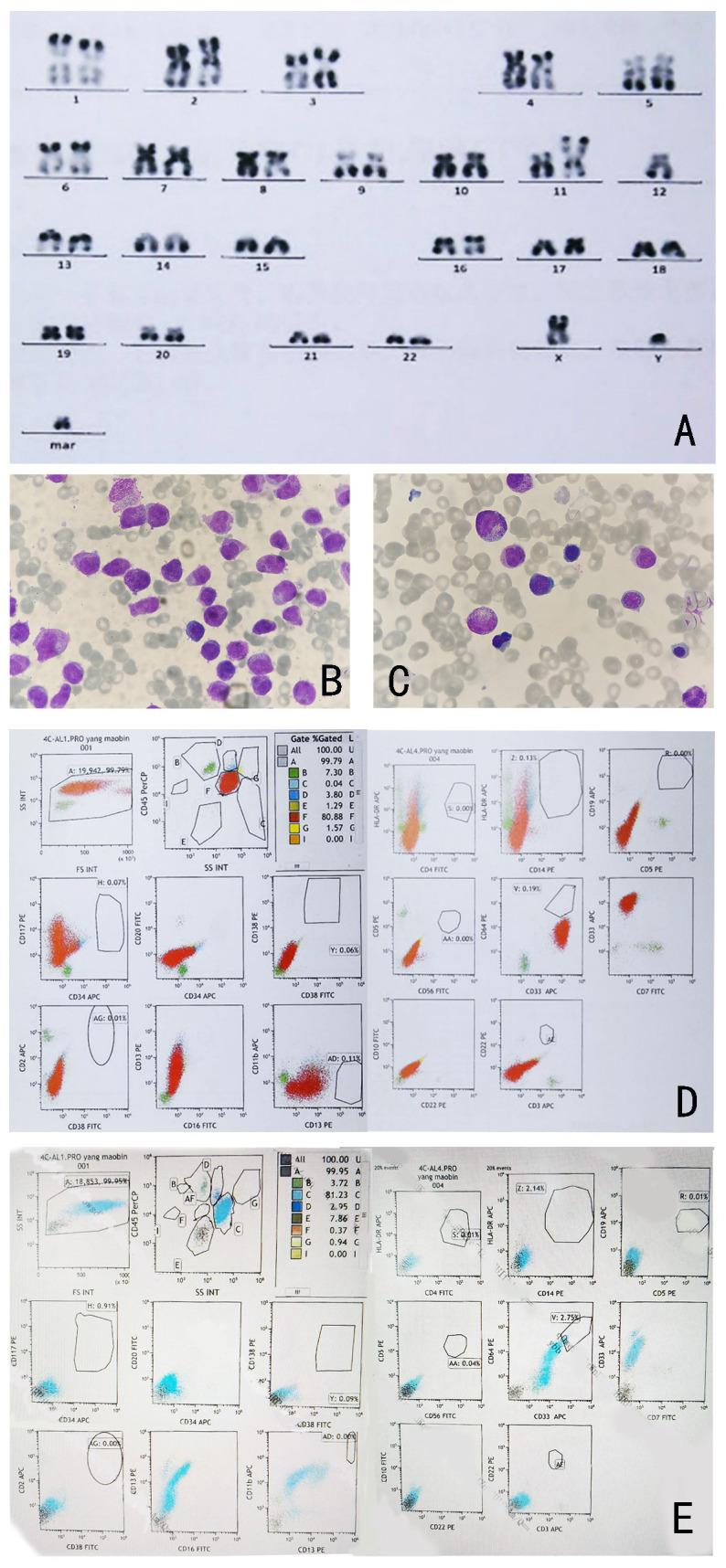
**(A)** Karyotype analysis shows 46, XY, t (11; 12) (p15; q13). **(B)** Morphology of bone marrow cells after IA and ATRA regimen. **(C)** Bone marrow morphology at complete remission after one cycle of azacitidine treatment (Wright-Giemsa-stained bone marrow smear, ×1000). **(D)** Immunophenotypic analysis results of bone marrow cells at diagnosis. **(E)**: Immunophenotypic analysis results of bone marrow cells at complete remission.

**Figure 3:**
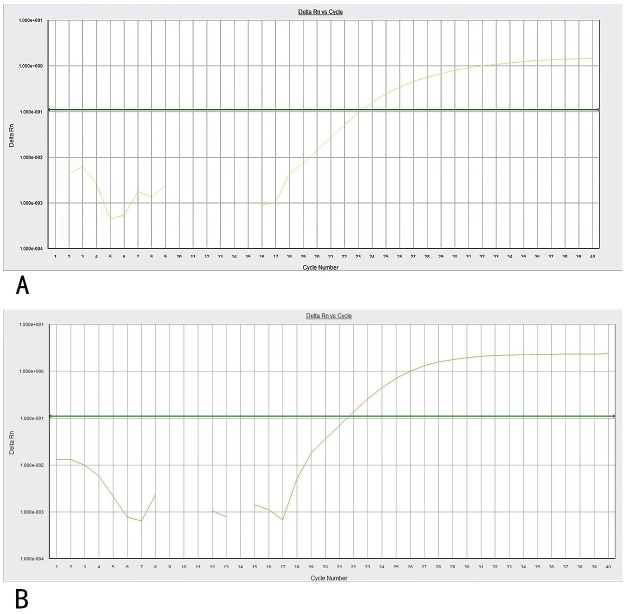
**(A)** PCR amplification curves of NUP98/RARG. **(B)** PCR amplification curves of ABL genes. Specific primers and probes were used for NUP98/RARG and ABL gene amplification.

Three weeks later, the patient was transferred to our hospital with myelosuppression and persistent fever. At the time of admission, blood tests showed hemoglobin, 59 g/L (reference range, 130–175 g/L); platelets, 26 × 10^9^/L (reference range, 125–350 × 10^9^/L); white blood cell count, 3.41 × 10^9^/L (reference range, 3.5–9.5 × 10^9^/L); prothrombin time, 15.9 s (reference range, 11–14.5 s); activated partial thromboplastin, 43.5 s (reference range, 28–45 s), fibrin degradation products (FDP) >150.00 ug/mL (reference range, < 5.00 ug/mL); fibrinogen, 3.56 g/L (reference range, 2.00–4.00 g/L); and D-dimer concentration > 20.0 ug/mL (reference range, < 5.00 ug/mL) (**Figure 1**). BM cytomorphology still supported APL (**Figure 2B**). Karyotype analysis of the chromosomes was 46, XY, der (11) t (11;12) (p15;q13), -12, +mar [15] (**Figure 2A**). The leukemia cellular immunophenotype was positive for CD117, CD13, CD33, MPO, and CD45^dim^ and negative for CD34, CD38, HLA-DR, CD11b, CD14, and other T- or B-lymphoid lineage markers (**Figure 2D**). Positive NUP98/RARG fusion gene (**Figure 3**) and mutations were detected in several genes at various frequencies including WT1 (variant allele fraction, VAF, 39%), TET2 (VAF, 5.1%), ARID1A (VAF, 45.9%), and KDM6A (VAF, 23.5%). The patient highest temperature on admission was 39.2°C, and the COVID-19 nucleic acid test was positive. The patient was administered paxlovid as antiviral treatment and other anti-infection treatments. One week later, the COVID-19 nucleic acid test result was negative and the patient still had an intermittent fever. Blood metagenomic next-generation sequencing (mNGS) and blood cultures were performed. Blood cultures were negative, and mNGS identified *Escherichia coli*. The patient developed abdominal pain, and an abdominal computed tomography (CT) scan showed acute pancreatitis. His pancreatic enzyme levels were elevated, with amylase and lipase levels 806 IU/L (reference range, 30–180 IU/L) and 2310 IU/L (reference range, 23–300 IU/L), respectively. The patient was treated to inhibit pancreatic enzymes. Given his recent infection and poor performance status, the patient was unable to tolerate intensive chemotherapy. On June 13, the patient was treated with 75 mg/m^2^ azacitidine. However, he developed sudden dizziness, fever (38.0 °C), and blood pressure drop (73/45 mmHg) after two days. He was empirically placed on imipenem/cilastatin and tigecycline, administered fluid resuscitation, and started noradrenaline. When the patient’s vital signs were stable and the infection was controlled, 135 mg of azacitidine was continued for five days.

**Figure 4:**
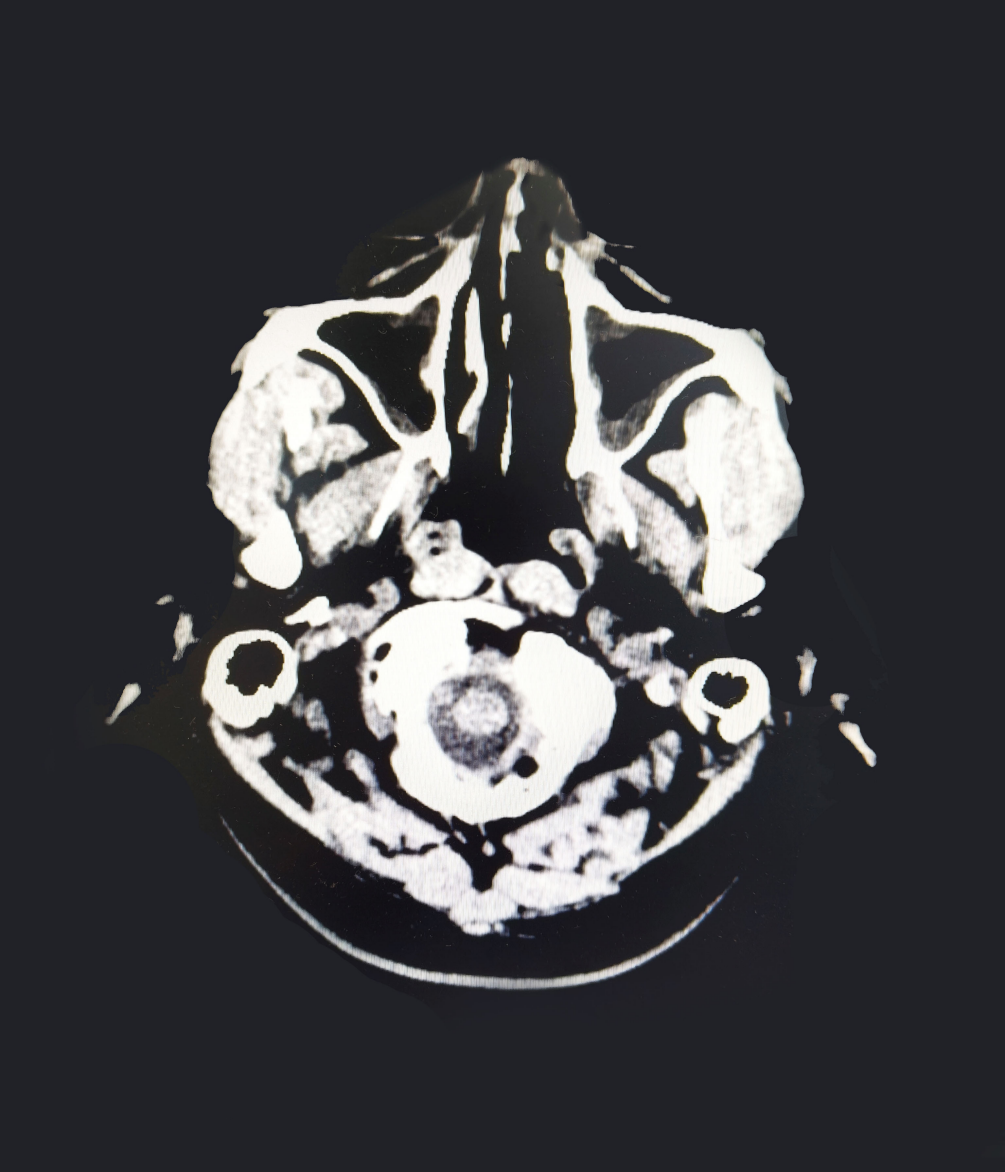
Paranasal sinus CT showing high density in the left nasal cavity.

Subsequently, the patient underwent craniocerebral + paranasal sinus CT for headache, which showed abnormal high-density foci in the brain and high density in the left nasal cavity with possible secretion (**Figure 4**). A consultation with the otolaryngology department suggested a diagnosis of rhinocerebral mucormycosis. The patient’s symptoms were relieved after antifungal administration and other treatments (the patient underwent sinonasal debridement at another hospital three months later; the postoperative pathomorphology was considered mucormycosis).

In July, BM aspirate morphology confirmed complete remission (CR) with negative minimal residual disease through flow cytometry (FCM) (**Figures 2C, E**). The positivity rate for NUP98 gene rearrangements dropped to 10%. Seven-day therapy with 75 mg/m^2^ azacitidine was continued. One month later, NUP98 rearrangement was negative. A seven-day treatment with 75 mg/m^2^ azacitidine was continued.

In January 2024, after four cycles of azacytidine treatment, the patient underwent allogeneic hematopoietic stem cell transplantation. Four months after transplantation, the patient remained in CR and was negative for the NUP98-RARG gene fusion (**Table 1**).

**Table 1:** Laboratory results.

Date	05–15	06–06	07–20	08–24
BM morphology leukemic cells	97%	96%	0	0
Wbc (×10^9^/L)^a^	72.06	3.41	12.23	4.12
Hb (g/L)^a^	96	59	105	138
Plt (×10^9^/L)^a^	21	26	208	433
FISH: NUP98	/	92%	10%	0.6%
MRD^a^(FCM)	None	None	< 0.01%	< 0.01%

a Wbc, white blood cells; Hb, hemoglobin; Plt, platelets; and MRD, minimal residual disease.

## Discussion

Such et al. first reported NUP98-RARG AML with APL morphology and features of the t(11;12)(p15;q13) translocation (2). When the patient relapsed and underwent induction therapy again, ATRA was administered in addition to chemotherapy. Although the patient achieved CR, the patient’s primary parental cells showed that AML with the NUP98-RARG rearrangement was insensitive to ATRA *in vitro* (5). In a global study of RARG, the 16 patients who received ATRA + ATO induction therapy (≥ 14 days) showed resistance to the drug combination and subsequently received AML induction therapy (6). However, another *in vivo* experiment conducted in a mouse model revealed that cells transformed with NUP98/RARG fusion were extremely sensitive to ATRA treatment (7), but the results of the murine model do not necessarily reproduce in humans. In the present case, the sensitivity of the patient could not be definitively assessed because he did not receive ATRA + ATO alone. It is unclear why the RARG rearrangements resemble the APL phenotype. A patient with a positive EZH2-D185H but negative PML-RARA fusion gene exhibited an APL phenotype by down regulating RARA and RARG expression; dysregulation of the RARA and RARG genes may be responsible for AML with an APL-like phenotype (8). Owing to NUP98-RARG AML resistance, when the patient does not show PML/RARA rearrangement, combined chemotherapy should be administered during induction therapy rather than ATRA + ATO.

The apparent paradox of ATRA producing resistance *in vivo* and responsiveness *in vitro* is due to several factors, including relatively short *in vitro* culture assays, different genetic backgrounds, and the acquisition of additional mutations. Most patients with RARG rearrangements have additional mutations in WT1, TP53, TET2, and KRAS. The acquisition of additional mutations, such as the WT1 mutation, may render patients with recombinant RARG resistant to ATRA. Approximately 10% of patients with AML have WT1 mutations, whereas WT1 mutations are present in over 50% of RARG-rearranged patients (6, 9). WT1 mutations are most closely associated with induction failure (10). NUP98-RARG gene rearrangements and multiple epigenetic-related gene mutations, such as TET2, ARID1A, and KDM6A, were detected in this patient. This may explain the effectiveness of the demethylated drugs. Azacitidine, a hypomethylating agent, incorporates into DNA and RNA, leading to hypomethylation and direct cytotoxicity to abnormal hematopoietic cells in the bone marrow. This mechanism may explain its effectiveness in treating AML with NUP98-RARG rearrangement, particularly regarding the presence of multiple epigenetic-related gene mutations in this specific patient. These mutations may increase the vulnerability of leukemic cells to demethylating agents, thereby enhancing treatment efficacy.

In one case, azacitidine was used for maintenance therapy after transplantation, and a patient with AML and NUP98-RARG gene fusion presenting as APL was treated with induction and consolidation chemotherapy using venetoclax in combination with chemotherapy, and then achieved CR followed by haploidentical hematopoietic stem cell transplantation. Post-transplantation maintenance therapy with venetoclax and azacitidine remained in CR after two cycles (11). The application of demethylation drugs for NUP98-RARG gene fusion in AML requires further investigation.

ATRA is a potent and highly specific inducer of CD38 expression in human promyelocytic leukemia cells. ATRA-induced CD38 expression in myeloid cells is mediated through RARA (12). In flow cytometry, CD33^+^, CD13^+^, CD38^+^, CD64^+^, CD34^-^, and HLA-DR^-^ constitute the typical APL immunophenotype (13). In a global study on AML with RARG rearrangement, all patients were CD38^-^ and the present patient was also CD38^-^ (6). Most cases of this novel AML have morphological features similar to those of APL, making a timely diagnosis difficult. Therefore, some patients may receive ineffective ATRA therapy for long periods. Consequently, chemotherapy should be considered as soon as possible in CD38- and RARA-negative cases.

## Conclusions

This is the first report of a patient with RARG rearrangement who achieved positive outcomes following demethylation therapy after the failure of ATRA and AML induction chemotherapy (AML-like regimens). Our case suggests that azacitidine may be a viable therapeutic option for patients with AML and NUP98-RARG fusion.

## Data Availability

Due to ethical and privacy considerations, this case report involves only a single patient, and as such, detailed data cannot be shared. All data are kept confidential in accordance with ethical guidelines and privacy regulations.
